# Contribution of plant-induced pressurized flow to CH_4_ emission from a *Phragmites* fen

**DOI:** 10.1038/s41598-020-69034-7

**Published:** 2020-07-23

**Authors:** Merit van den Berg, Eva van den Elzen, Joachim Ingwersen, Sarian Kosten, Leon P. M. Lamers, Thilo Streck

**Affiliations:** 1 Institute of Soil Science and Land Evaluation, Biogeophysics, University of Hohenheim, Emil-Wolff-Straße 27, 70593 Stuttgart, Germany; 2 Department of Aquatic Ecology and Environmental Biology, Institute for Water and Wetland Research, Radboud University, PO Box 9010, 6500 GL Nijmegen, The Netherlands

**Keywords:** Carbon cycle, Wetlands ecology

## Abstract

The widespread wetland species *Phragmites australis* (Cav.) Trin. ex Steud. has the ability to transport gases through its stems via a pressurized flow. This results in a high oxygen (O_2_) transport to the rhizosphere, suppressing methane (CH_4_) production and stimulating CH_4_ oxidation. Simultaneously CH_4_ is transported in the opposite direction to the atmosphere, bypassing the oxic surface layer. This raises the question how this plant-mediated gas transport in *Phragmites* affects the net CH_4_ emission. A field experiment was set-up in a *Phragmites*-dominated fen in Germany, to determine the contribution of all three gas transport pathways (plant-mediated, diffusive and ebullition) during the growth stage of *Phragmites* from intact vegetation (control), from clipped stems (CR) to exclude the pressurized flow, and from clipped and sealed stems (CSR) to exclude any plant-transport. Clipping resulted in a 60% reduced diffusive + plant-mediated flux (control: 517, CR: 217, CSR: 279 mg CH_4_ m^−2^ day^−1^). Simultaneously, ebullition strongly increased by a factor of 7–13 (control: 10, CR: 71, CSR: 126 mg CH_4_ m^−2^ day^−1^). This increase of ebullition did, however, not compensate for the exclusion of pressurized flow. Total CH_4_ emission from the control was 2.3 and 1.3 times higher than from CR and CSR respectively, demonstrating the significant role of pressurized gas transport in *Phragmites*-stands.

## Introduction

Methane (CH_4_) produced in soils is to a great extent oxidized to CO_2_ before it reaches the atmosphere^1,2^. The proportion of CH_4_ oxidation depends on water table height^1–3^ and the presence or absence of vascular wetland plants^4,5^. Both factors influence the concentration of oxygen (O_2_) in the soil^6,7^ and the pathways by which CH_4_ is transported from the soil to the atmosphere^1,5^. There are three gas transport pathways: diffusion and ebullition from the soil, and plant-mediated transport via aerenchyma of roots and stems of vascular plants.

Diffusion is a relatively slow process, whereby a large part of the produced CH_4_ is oxidized when crossing the oxic upper layer of the water column or soil^2^. Ebullition occurs when gas builds up in a submerged soil and bubbles are formed. When bubbles are erupted episodically, the bubbles can rapidly pass through the water-saturated soil and water column above. Because this transport is fast, only a small part of the CH_4_ will be oxidized. In general, ebullition is affected by temperature, air pressure and water table height (influencing the pressure in the water column); however, it shows a high spatial and temporal variation and is hard to predict^2,5,8–10^. The contribution of ebullition to the overall CH_4_ flux ranges from a few percent^11^ to more than 50%^12^.

CH_4_ transport mediated by wetland plants occurs through aerenchyma, which has the physiological function to transport O_2_ into the roots. Often, more O_2_ is transported to the roots than is consumed, leading to O_2_ leakage into the rhizosphere^13^. Besides O_2_ transport into the soil, other gases (e.g. CO_2_ and CH_4_) can simultaneously be transported from the soil to the atmosphere^14,15^. This gas transport via aerenchyma tissue can occur via a diffusion gradient or by a pressure gradient that is built up by the plants^7,16^. Plants that transport gases via diffusion are, among others, *Carex rostrata*, *Oryza sativa*, *Scirpus lacustris* and *Peltandra virginica*^14,17^, whereas gas transport via convective through-flow due to pressure gradient is found in *Typha latifolia*, *Typha angustifolia*, *Nymphaea odorata*, *Nuphar luteum*, *Nelumbo nucifera*, *Nymphoides peltata* and *Phragmites australis*^7,14^.

**Figure 1:**
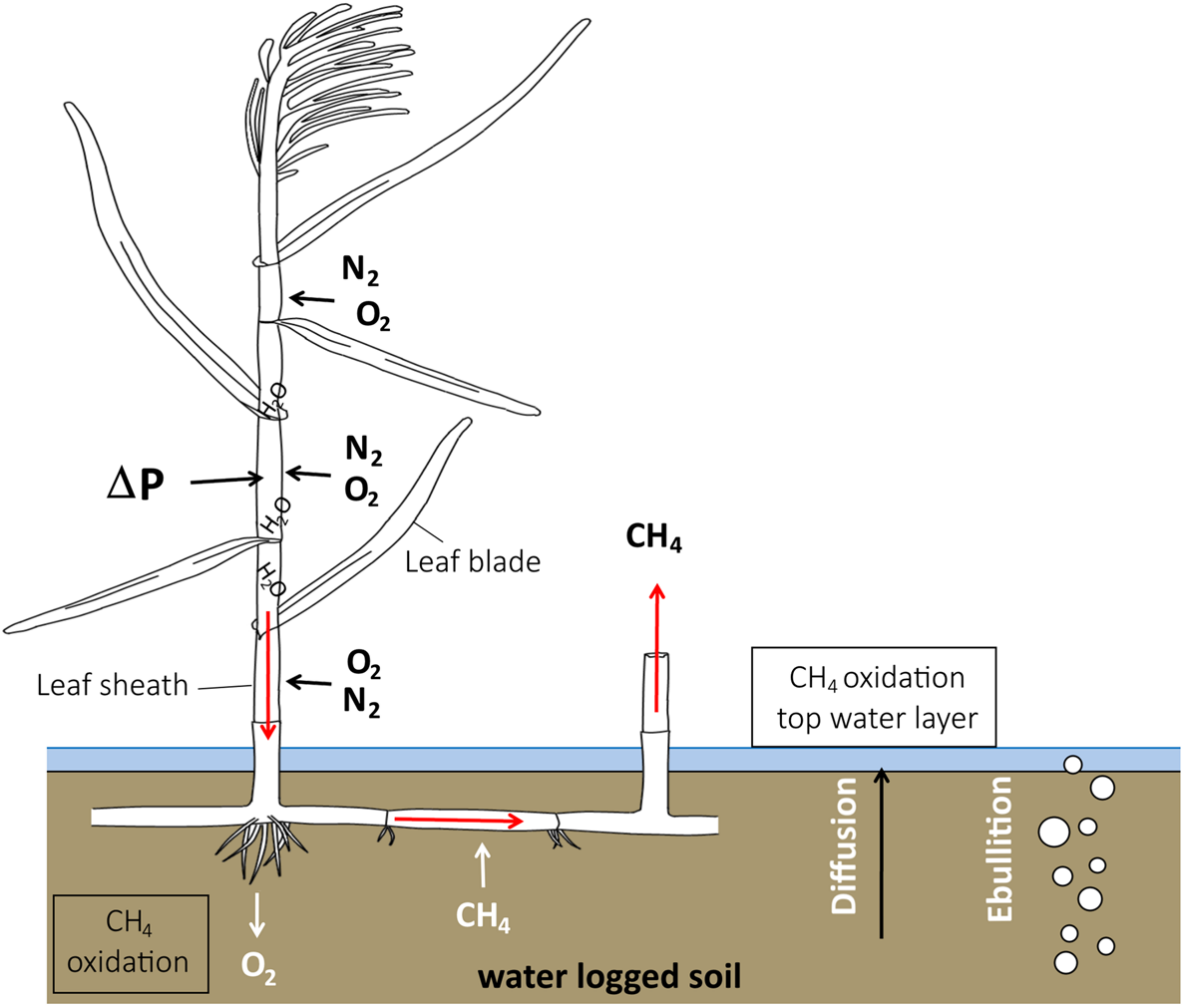
Schematic overview of the humidity induced convection inside *Phragmites* plants. N_2_ and O_2_ are transported through stomata of the leaf sheath, following the diffusion gradient. A higher pressure inside the stem is created in living plants (ΔP), which creates an airflow towards the rhizome and goes back to the atmosphere via old or broken stems (red arrows). O_2_ is transported to the soil and CH_4_ that diffuses into the rhizome will be transported to the atmosphere. On the places where O_2_ and CH_4_ are present together, CH_4_ oxidation occurs.

Gas transport in *Phragmites australis* (Cav.) Trin. ex Steud. (common reed) is highly effective. It builds up a humidity-induced pressure gradient within the leaf sheaths (the part of the leaf that encircles the stem) that leads to an airflow from the leaf sheaths towards the rhizomes, which is vented via old and broken stems^6,16, 18,19^. This transport mechanism is more than five times faster than diffusion^3^ and is regulated by the pores (stomata) in the leaf sheaths. These stomata do not transport gas caused by pressure differences, but allow gas transport by diffusion. Due to higher humidity in the internal culm of *Phragmites*, O_2_ and N_2_ concentrations inside the plant are diluted. Therefore, O_2_ and N_2_ are transported along the concentration gradient from the atmosphere into the sheaths, increasing the internal pressure (see Fig. 1). Since this process depends on stomatal conductance, humidity induced convective flow starts after sunrise when the stomata open, reaches an optimum around noon when photosynthetic rates are highest and then decreases till sunset^3^. Therefore, this diurnal cycle is also observed in CH_4_ fluxes from *Phragmites* wetlands during the growing season^4,17,20,21^. On the one hand, *Phragmites* transports O_2_ into the soil, which leads to higher oxidation rates of CH_4_ and can also be expected to reduce methanogenesis. On the other hand, transport of CH_4_ from the soil to the atmosphere through the plant is facilitated, bypassing the oxic soil and water layer. Grünfeld & Brix^4^ showed a 34% decrease in CH_4_ emission after addition of *Phragmites* to a submerged organic soil. In contrast, Hendriks et al.^5^ found that vascular plant presence (among others *Phragmites* and *Thypha latifolia*) leads to higher methane emissions, but depends on the water table height.

Since the findings in literature are ambiguous, the following questions remain: (1) how important is plant-mediated gas transport in *Phragmites* compared to the other CH_4_ transport mechanisms (diffusion and ebullition from the soil); (2) how does *Phragmites* influence diffusive and ebullition fluxes; and (3) does the presence of *Phragmites* stands lead to an overall increase or decrease of CH_4_ emission? To study this, a field experiment within a measuring period of three weeks during the growing season was set-up in a large reed area of a minerotrophic peatland. To quantify the importance of plant-mediated CH_4_ transport, we compared fluxes measured with chambers from control *Phragmites* plots with plots where *Phragmites* stems were clipped to exclude pressurized gas transport through the plant. To exclude any gas transport through plants, we also measured CH_4_ fluxes from plots where *Phragmites* stems were clipped and sealed. Ebullition from the soil was determined as well, to assess the relative contribution of all gas transport pathways of CH_4_ from a reed fen.

In addition to the experiment, we were interested to see if the chamber fluxes from the control plots were representative for the total system. Therefore, we made a comparison between CH_4_ fluxes measured with the chamber method and with the eddy covariance method.

## Materials and methods

### Study site

The study was conducted in the Federseemoor (48.092°N, 9.636°E), a peatland of 30 km^2^ located in the region Upper Swabia in southwest Germany. This peatland has developed via natural terrestrialization from a proglacial lake after the last ice age. As a result, the surface area of the lake declined from 30 to 12 km^2^. Between 1787 and 1808, the lake was further reduced to a size of 1.4 km^2^ by drainage activities. The newly gained land of 11 km^2^ was used as pasture but turned out to be unprofitable due to the recurring high water table. Nowadays it is a nature conservation area, mainly consisting of fen (see van den Berg et al.^21^ for a vegetation map). The lake Federsee is completely surrounded by reed vegetation (*P. australis*), with a total area of 2.2 km^2^ and a density of around 70 living shoots and 75 dead stems per m^2^. During the measurement period (7–10 June) the *Phragmites* plants were 1.2 m high. This is half their maximum height, which is reached at the end of July. The high density of *Phragmites* and lack of other species in the reed belt result from high nutrient concentrations due to wastewater input to the lake since 1951. After 1982, the input of untreated sewage water was stopped, which reduced the nutrient concentrations. Only since 2006 has there been a significant improvement in water quality, and after 2008 the lake water became clear again. The field experiment was installed in the middle of the reed area at around 70 m distance from an eddy covariance (EC) tower, which has been running since March 2013^21^. In a radius of at least 200 m around the EC tower, the vegetation is dominated by *Phragmites* (see van den Berg et al.^21^), meaning only reed dominated the measured EC footprint.

### Field experiment

Nine plots of 2 m × 2 m were prepared for three treatments with three replicates: (1) clipped reed (CR), to exclude the pressurized flow in the plants; (2) clipped and sealed reed (CSR), to exclude any exchange via plant stems; and (3) control where reed was not manipulated. In the CR and CSR treatments, living and dead reed stems were clipped to about 10 cm above the water table. In the CSR treatment the clipped reed stems was sealed with an acrylic sealant. Since rhizomes connect plants over longer distances, plots were isolated by cutting rhizomes from the reed plants around each plot to a depth of 50 cm, to avoid gas exchange with the surrounding area. The period between preparation of the plots and measurements was minimized (1–2 days) to reduce possible side effects, such as change in substrate availability for methanogens. One day before the first measurement, the water table rose about 20 cm in the whole field, flooding the prepared sealed stems of one plot already prepared for the CSR treatment. Nevertheless, since no gas exchange is expected from the sealed stems, this plot was still included in the experiment. CH_4_ and CO_2_ diffusive fluxes from the soil and plant-mediated fluxes were measured with transparent flow through chambers. Pore water was extracted to analyze the effect of the reduced/excluded gas exchange by the plants on soil chemistry. In each plot ebullition was measured as well (see below).

### Diffusive and plant mediated CH_4_ flux

On 7, 9 and 10 June 2016 between 07:00 and 18:00, the gas fluxes of each treatment were alternately measured. Per day, only one of the triplicates per treatment was measured. CH_4_ fluxes were measured in the middle of the plots with transparent chambers with a diameter of 50 cm. One chamber was 2 m high and was on the control plots. Two chambers were 1 m high and used on the CR and CSR plots. The 1-m chambers were equipped with a small fan of 8 cm × 8 cm that had a flow capacity of 850 l min^−1^; two fans were installed in the 2-m chamber. Each day one replicate of every treatment was measured, to be able to capture the diurnal cycle for each plot and to minimize disturbance by translocating the chambers. The chambers were connected with 8 m tubing to a multiport inlet unit attached to a fast greenhouse gas analyzer (GGA) with off-axis integrated cavity output spectroscopy (GGA-24EP, Los Gatos Research, USA) measuring the concentration of CH_4_ and CO_2_ every second. Every 5 min, the multiport switched between the three chambers, allowing air from each chamber to be alternately pumped through the GGA with a pumping rate of 300 ml min^−1^ and resulting in four flux measurements per plot per hour (~ 35 measurements per plot per day). The withdrawn air from the chamber was replaced with ambient air through an opening in the chamber. After 1–2 h of continuous measurements, the chambers were ventilated by lifting the chambers to fully replace inside air with ambient air. After 15 min, the chamber was put back and measurements continued. Since it takes a long time before the chamber CH_4_ gets to equilibrium with the water column, 1–2 h of increasing CH_4_ concentration in the chamber will have little effect on the measurement accuracy of the CH_4_ flux (in contrary to the CO_2_ flux)^22^. Nevertheless, we used only data from the first 30 min after ventilating to calculate the diffusive flux (five measurements per plot per day), since this is the period where temperature and humidity inside the chamber resemble outside conditions most closely. Only for the comparison between eddy covariance fluxes and chamber fluxes on the control plots we did use data from the whole measurement period.

The concentration for every measurement point was corrected for the change in concentration caused by the inflow of ambient air with known CO_2_ and CH_4_ concentrations (measured by the EC station) and outflow of chamber air (both with a flow rate of the pump speed of the Los Gatos). The slope of the corrected chamber concentrations over a 4 min period within the 5 min measurement was used to calculate the flux and was checked for non-linear fluctuations due to e.g. ebullition. Fluxes corresponding to an average chamber concentration of > 100 ppm CH_4_ were discarded, because of the GGA’s detection limit. In total 11% of the fluxes were discarded.

### Ebullition

In each plot ebullition was measured by catching bubbles from a fixed surface with an ebullition trap^10^, composed of a 20 cm diameter funnel, to which a glass bottle of 300 ml was attached. The bottles were filled with water from the site and the ebullition trap was installed under the water table on 8 June and carefully anchored between reed stems (no open endings of stems were below the trap) on the soil surface around 0.55 m below the water surface. Bubbles were captured in the glass bottle for 18 days, after which the bottles were removed and gas samples were taken in the field. The total volume of ebullition gas was determined and the concentration of CH_4_, CO_2_ and N_2_O were measured by gas chromatography (7890B GC, Agilent Technologies, USA) in the lab.

### Environmental variables

In each chamber, temperature and radiation were measured with a temperature/light sensor (HOBO Pendant data logger, Onset Computer Corporation, USA) logging at an interval of 30 s. Every minute soil temperature was measured in each plot in the upper 0–0.05 m with a Soil Water Content Reflectometer (CS655, Campbell Scientific Inc., USA) around 0.56 m below the water table. Air temperature, air relative humidity (HMP155, Vaisala Inc., Finland) and incoming and outgoing shortwave and longwave radiation (CNR4, Kipp & Zonen Inc., The Netherlands) were measured at a height of 6 m close to or at the EC station. Groundwater table was continuously measured with a water level pressure sensor (Mini-Diver datalogger, Eijkelkamp Agrisearch Equipment Inc., The Netherlands) placed at 1.45 m depth in a 2-m long filter pipe that was placed 1.60 m into the soil. Data were recorded at a 30 min interval.

### Pore water sampling and analysis

To see if the treatments had any effect on the methane production, pore water samples were analyzed. At two locations in each plot, pore water was extracted anaerobically with ceramic cups (Eijkelkamp Agrisearch Equipment Inc., The Netherlands). Pore water from 10, 20, 30 and 50 cm depth was collected by vacuum suction in syringes and transported to the lab. In the lab, pore water was diluted with a ratio of 1:3. Dissolved organic carbon (DOC) concentration was measured with a Dimatoc 100 DOC/TN-analyzer (Dimatec, Germany). A second pore water sample was taken in vacuumed 13 ml exetainers with 3 g of NaCl. The concentration of CH_4_ in the headspace of these exetainers, representing the CH_4_ concentration in pore water, was determined on a HP gas chromatograph (Hewlett Packard, USA). A third pore water sample was fixed with 0.2% 2.2-bipyridin in 10% CH_3_COOH buffer in the field to determine Fe(II) measuring photometrical absorption at 546 nm in the lab.

### Eddy covariance

The EC tower was located at a distance of around 70 m from the prepared plots. The tower was 6 m high and consisted of a LI-7700 open path CH_4_ gas analyser (LI-COR Inc., USA), a LI-7200 enclosed path CO_2_/H_2_O gas analyser (LI-COR Inc., USA) and a WindMaster Pro sonic anemometer (GILL Instruments Limited Inc., UK). Molar mixing ratio/mass density of the gases and wind speed in three directions were measured at a frequency of 10 Hz. Fluxes were calculated for an averaging interval of 15 min with the software EddyPro version 6.1.0. For more detailed information about the set up and calculations of the fluxes, see van den Berg et al.^21^.

### δ^13^C measurements

CH_4_ oxidation and transport lead to isotopic fractionation of δ^13^C of CH_4_^23^. The difference between δ^13^C of the CH_4_ present in the soil and the CH_4_ emitted to the atmosphere may therefore reveal the importance of both methane oxidation and the different emission pathways.

The δ^13^C of CH_4_ tends to be much lower than the natural abundance in organic compounds, because methanotrophic prokaryotes prefer the lighter ^12^CH_4_ to ^13^CH_4_ thereby increasing the δ^13^C of CH_4_. Diffusion rates for ^12^CH_4_ are higher than for ^13^CH_4_^14^ decreasing the δ^13^C of the emitted CH_4_^23^. Although ^13^C enrichment (compared to produced CH_4_) has been found in internal spaces of plants due to CH_4_ oxidation^14^, the fractionation at the plant-atmosphere surface reduces the δ^13^C by about 12–18‰ due to the faster transport rate of ^12^CH_4_, which makes that emitted CH_4_ can have a lower fraction of δ^13^C than the produced CH_4_. Differences in δ^13^C between sediment and overall emission are larger for plants with diffusive internal gas transport than for plants with convective gas transport^23^.

Since fractionation of CH_4_ emitted through ebullition in shallow waters is negligible, these gas bubbles can be used to know the isotopic composition of CH_4_ produced in sediment^23^. We therefore compared the δ^13^CH_4_ signature of ebullition gas with the signatures of CH_4_ from the chambers. Gas samples from the chamber were taken when the CH_4_ concentration was at least 10 times the ambient concentration, from each plot in the afternoon. The δ^13^CH_4_ signature was measured with an isotope-ratio mass spectrometer Delta plus XP (Thermo Finnigan, Germany).

### Statistics

Chamber fluxes were measured at different times of the day, which means that environmental variables like temperature and radiation were varying. To be able to compare the different treatments without the variation resulting from environmental conditions, an analysis of covariance (ANCOVA) was conducted with the environmental variables as covariables. For the analysis, the data of the different measurement days were pooled together per treatment. The residuals of the model were normally distributed. With the parameters of the ANCOVA model, average fluxes were calculated with average environmental variables for the period ebullition was measured (8–27 June), to be able to compare the chamber fluxes with ebullition.

To test if the means of the ebullition measurements or pore water concentrations were different between the treatments, an analysis of variance (ANOVA) test was performed with Fishers’s Least Significant Difference (LSD) post hoc test to find the specific differences between the treatments.

## Results

### Environmental conditions

May and the first week of June were very wet with extreme rain events (up to 45 mm day). This caused the water table to rise to 55 cm above the surface, while normal fluctuations of the water table are between − 2 and 40 cm with an average of 8 cm above surface. During the measurement days rainfall was little to zero (see Table 1). Only on 9 June the weather was more cloudy with some rain in the morning. Ambient daily average temperature was close to the monthly average of 18.1 °C.

**Table 1:** Average of environmental variables during the measurement period 07:00–18:00 of each measurement date.

Date	Ambient temp. (°C)	Soil temp. 5 cm (°C)	Water table (cm above surface)	Rainfall (mm day^−1^)	Incoming radiation (W m^−2^)
07-06-2016	22.3	13.4	53.8	0	648
09-06-2016	15.6	13.7	56.0	2.8	345
10-06-2016	18.6	13.8	56.7	0	606

### Pore water

**Figure 2:**
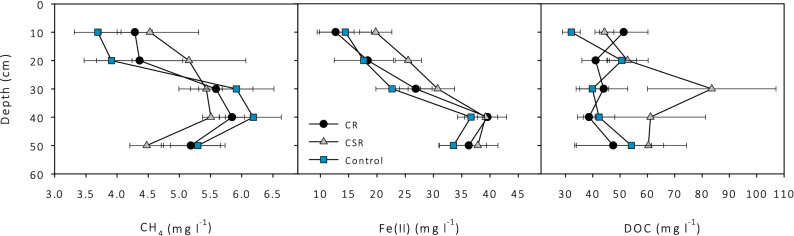
Average dissolved CH_4_ (left), ferrous iron (Fe(II)) (middle) and dissolved organic carbon (DOC) (right) in pore water at 10–50 cm depth for the treatments clipped reed (CR), clipped and sealed reed (CSR) and the control. Error bars denote the standard error from 6 measurements.

Most roots are concentrated at 30 cm depth (personal observation). From this depth and lower, Fe is reduced to Fe(II) and CH_4_ production is enhanced and/or CH_4_ oxidation reduced, given the increase in CH_4_ concentration at this depth (see Fig. 2). Concentrations of elements in pore water show small (not significant) differences in CH_4_ and Fe(II) concentration between the treatments. A significant increase is found in DOC concentrations for the CSR treatment compared to the control (*p* < 0.05) at 30 cm depth.

### Comparison eddy covariance versus chamber fluxes

**Figure 3:**
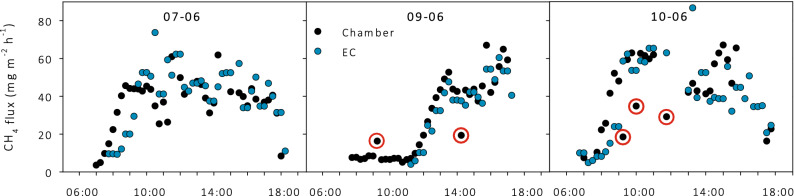
Fluxes measured with chambers on three control plots (i.e. plots with unmodified reed stands), one plot measured per day, and with the eddy covariance (EC) method over the same days. The red circles indicate chamber data that deviate from EC data pattern and correspond with the first measurement after ventilating.

During the experiment, the CH_4_ fluxes measured by the EC and the chamber method on the control plots show a similar data range and show the same diurnal pattern (Fig. 3). The later increase in CH_4_ flux in both EC and chamber data on 9 June compared to the other days is most probably due to the low radiation by cloud cover and rain in the morning. From 9 June, there are no EC data available between 7:00 and 11:00 due to a rain event, which disturbed the functioning of the open-path CH_4_ sensor. Chamber fluxes data that did not match the EC flux pattern well appeared to originate from the first measurement after ventilating the chamber (see Fig. 3). Therefore, all first measurements after ventilating were discarded in further analyses.

These results show that side effects of the chamber like temperature increase or high relative humidity did not affect the CH_4_ flux (and thus gas transport through the plant) much, which makes the comparison between the control and other treatments reliable.

### Diffusive and plant-mediated fluxes

**Figure 4:**
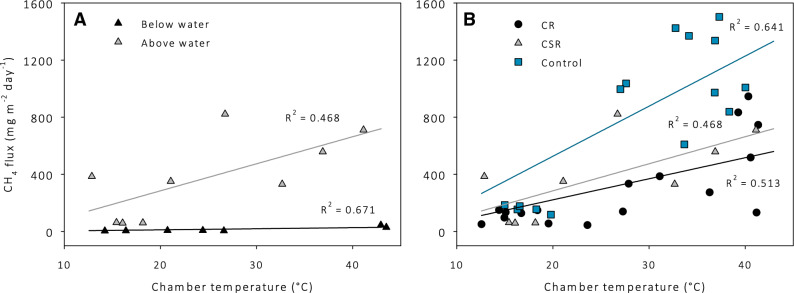
CH_4_ flux in relation to chamber temperature. (**A**) The treatment clipped and sealed reed (CSR), in black the measurements in the plot with the stems below water (7 June) and in grey the plots with stems above water (9 June, 10 June). (**B**) All treatments (clipped reed (CR), clipped and sealed reed (CSR) and control) excluding the measurements from CSR with stems below water. The linear regression models are all significant (*p* < 0.001).

All treatments show a diurnal cycle that correlates well with the inside air temperature of the chamber. However, the stems of the CSR plot measured on 7 June were flooded. This clearly affected the CH_4_ flux and the relation with chamber air temperature (Fig. 4), compared to the other measurements in the CSR treatment. Flooding of the sealed stems resulted in a further decrease of the gas flux. Due to the different conditions, these data were excluded from further data analysis. The ANCOVA analysis gives a significant result for the interaction chamber temperature * treatment (*p* < 0.05, F = 84.2), which means that temperature has a significantly different effect on CH_4_ flux between the treatments. This interaction is used in the model, with the results given in Table 2. The regression models for each treatment (the results of the ANCOVA analysis) are plotted together with the measured fluxes in Fig. 4.

**Table 2:** Descriptive statistics and slopes (beta) with significant levels for the different treatments in the ANCOVA model.

Treatment	N	Mean	SD	Beta interaction	Sig beta
CR	17	300.5	289.6	14.7	0.000
CSR	9	370.7	649.4	19.0	0.000
Control	15	792.2	508.7	35.1	0.000

The interaction is (chamber temperature − 5) * treatment. N is amount of data points.

All regression lines in the model are forced through an intercept of 5 °C, assuming that there is no significant microbial activity below this temperature^24^. This was done by subtracting 5 degrees from the measured temperature and excluding an intercept in the model. With this model, including the three regressions, the F value is 84.2 (*p* < 0.001) and the effect size (η^2^) 0.869. The control treatment showed the highest flux and CR treatment the lowest (Table 2). The slope of the control treatment is about twice as high as that of the other treatments (*p* < 0.001), but the slopes of CR and CSR are not significantly different from each other (*p* = 0.359).

### Ebullition

Total volumes of trapped ebullition gas from the soil differed between the treatments and control: CR and CSR have a 2–3 times higher release of ebullition gas than the control (76 ml m^−2^ day^−1^). The difference between CSR and control is significant (*p* < 0.05). For the CH_4_ volume both treatments are significantly different from the control (*p* < 0.05), but not significantly from each other. In both CR and CSR treatments, CH_4_ is clearly dominating ebullition: the percentage of CH_4_ in the ebullition gas was around 4 to 5 times higher in the CR (51%) and CSR (71%) treatments compared to the control (13%). Zero (CR and CSR plots) or very low amounts (0.6 ml m^−2^ day^−1^) (control plots) of N_2_O were found in the ebullition traps. Besides CH_4_ and CO_2_, ebullition gas could consist of nitrogen gas and water vapor, which were not measured.

### Relative contribution different flux pathways

**Figure 5:**
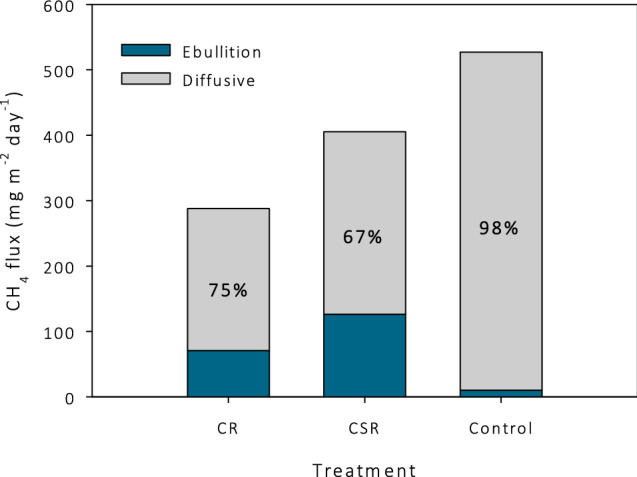
Total CH_4_ flux, consisting of ebullition and diffusive flux corresponding to a temperature of 14.7 °C (average ambient temperature between 7 and 27 June) for the treatments clipped reed (CR), clipped and sealed reed (CSR), and control. The contribution of the diffusive flux to the total flux is given in percentages.

To compare the ebullition flux with the diffusive/plant mediated flux, we calculated the average daily chamber flux based on the relation of CH_4_ flux with temperature from the ANCOVA model (Table 2). The fluxes were calculated for a temperature of 14.7 °C, which is the daily average ambient temperature from 8 to 27 June (the period in which ebullition gas was captured), and resulted in an average flux of 517 (control), 217 (CR) and 279 mg m^−2^ day^−1^ (CSR). The fraction of ebullition from the total flux is 13 to 16 times higher in the CR and CSR treatments than in the control (Fig. 5).

### δ^13^C change from soil CH_4_ to emitted CH_4_

In all treatments the CH_4_ sampled in the chamber show lower δ^13^C than CH_4_ from ebullition (Table 3). The largest depletion was found for CR, followed by CSR and control.

**Table 3:** δ^13^C measured in ebullition and chamber flux.

Treatment	Chamber flux		Ebullition		δ^13^C change
	δ^13^C	SE	δ^13^C	SE	
CR	− 62.21	1.44	− 50.93	1.59	− 11.28
CSR	− 63.05	1.53	− 55.95	0.87	− 7.10
Control	− 60.35	1.29	− 55.44	1.46	− 4.91

The difference is a measure of the fractionation due to oxidation or gas transport pathways.

## Discussion

The total CH_4_ flux (chamber + ebullition) decreased by 45% and 23% when we clipped the reed (CR) and clipped and sealed the reed (CSR), respectively, compared to intact reed (control) (see Fig. 5). The contribution of ebullition to the total flux increased much by clipping: 2% in the control plots compared to 24% in CR and 37% in CSR (see Fig. 5). van der Nat et al.^17^ found a lower CH_4_ flux from water saturated bare soil compared to *Phragmites*-vegetated soil, with a difference of 75%. They also found that in bare soil (compared to *Phragmites* vegetated soil) ebullition was the main gas transport pathway, while in a *Phragmites*-vegetated soil more than 98% of the CH_4_ was transported through the reed. Our findings, however, contradict the findings of Grünfeld & Brix^4^. They showed in an experiment a 34% lower CH_4_ emission with *Phragmites* in a submerged organic soil compared to the same soil without *Phragmites* and argued that methanogenesis is reduced and CH_4_ oxidation increased because of the transport of oxygen by *Phragmites* into the rhizosphere. In the soil without reed, the gas transport would be dominated by ebullition. The reason for the different findings of Grünfeld & Brix^4^ compared to our data, could be due to differences in experimental set up. Their experiment was conducted with single *Phragmites* seedlings (6 months old) and CH_4_ flux was measured 9 weeks after planting. This means that there was no rhizome network present as in a developed reed bed. Because CH_4_ is taken up by the rhizomes and transported upwards, the amount of CH_4_ transport in *Phragmites* seedlings would be lower than at our site. Seemingly, the oxygen transport to the soil was not much limited by the undeveloped rhizome network of the seedlings. In our study CR and CSR reduced or excluded plant-mediated gas transport, but fluxes are not directly comparable to those from bare soil.

In CR, roots and clipped stems still allowed gas transport via diffusion, but not via pressurized flow as in the control plots. The clipped stems act as chimneys that connect deeper soil layers to the atmosphere. In line with this, Greenup et al.^25^ found that CH_4_ flux from *Sphagnum* vegetation increased after inserting glass tubes into the soil. CR only showed slightly lower chamber flux than CSR (217 vs 279 mg m^−2^ day^−1^) (see Fig. 5), indicating that sealing the stems hardly affected the chamber CH_4_ flux (disregarding ebullition). Only the CSR plots with stems below water (see "Material and methods" section) showed a clear reduced CH_4_ flux compared to plots with stems above water. This suggests that our sealing method was not fully effective. Possibly gas could still escape from, for example, cracks in the stem. Despite this chimney function, we found that diffusive fluxes from clipped reed were two times lower compared to the control (see Fig. 4). We found that despite the higher ebullition from CR and CSR treatment, the increase does not compensate for the excluded pathway via the pressurized air flow. This could indicate a significant role of pressurized flow in intact reed beds.

The total CH_4_ flux could be overestimated in our data, and should therefore be interpreted with care. Ebullition could exists of episodical outbursts and/or a steady flow (small bubbles continuously released from the soil)^11^. Steady ebullition cannot be separated from diffusion in chamber flux measurements since the flow is constant, but is at the same time captured in bubble traps. So it could be that we double counted for this steady ebullition. It is hard to say if steady ebullition occurred or how much it contributes to the total flux. There is little scientific literature available that describes the prediction or characteristics of this type of gas flow. The conclusions about the increase in ebullition by clipping would, however, not change. And since ebullition is higher in the CR and CSR treatment, the double counting would be higher in the treatments as well, meaning that the difference in the total CH_4_ flux of the control vs treatments would only increase.

Another effect that we cannot quantify is the change in root exudates by cutting the reed. Root exudates are a substrate for methanogens and are expected to decrease by clipping due to the exclusion of photosynthesis. The change in photosynthates can occur within several hours, although a peak response of CH_4_ emission can be expected after several days^26,27^. The possible decrease in photosynthates did not noticeably increase DOC or CH_4_ concentrations in the pore water (see Fig. 2) and we therefore expect it had little influence on the fluxes.

The δ^13^C signature change we found shows the difference in the gas transport mechanism between clipped and unclipped *Phragmites* (see Table 3). Without considering δ^13^C signature change due to gas transport, emitted CH_4_ is expected to be enriched with ^13^C compared to produced CH_4_, since part of the CH_4_ is oxidized. This would result in a positive δ^13^C signature change, that we expected to be highest in unclipped *Phragmites*. We found the opposite: the δ^13^C depletion was larger in the CR and CSR treatments (− 11‰ and − 7‰ respectively) than in the control (− 5‰). This change in δ^13^C signature in CR and CSR is in the same range as in CH_4_ emission from plants with diffusive gas transport^14^. That clipped vegetation can be considered as plants with diffusive gas transport, is in line with the findings of Greenup et al.^25^. They did not find a significant difference between CH_4_ flux from clipped stems of *E. vaginatum* above the water table and from unclipped vegetation. Gas transport in *E. vaginatum* is known to be due to diffusion. The lower fractionation of CSR compared to CR treatment can be explained by the fact that gas transport through the stems is partly limited due to the sealing in the CSR treatment. Chanton^23^ compared δ^13^C change from soil to atmosphere from different wetland plants and found, on average, a smaller change for plants with convective transport than for those with diffusive transport. That corresponds to our results. Assuming gas transport in clipped reed to be diffusive, we can summarize that pressurized CH_4_ transport in intact reed leads to a CH_4_ emission two times higher than clipped reed with diffusive transport through stems.

Our chamber measurements from the control plots show diurnal patterns very similar to the EC measurements, with low fluxes in the morning and simultaneous increases when light intensity and temperature increased. Such a similarity in fluxes is not often found in comparisons between the two methods^5,28–31^. Our results can be explained by the very homogeneous EC footprint of our site in regard to vegetation and landscape development (see *Study site*). Thereby, ebullition contributes for a small amount to the total flux, this leads to a more constant flux without abrupt and random emission peaks of CH_4_. The highest discrepancy between chamber and EC fluxes were found within 10 min after ventilating. Lifting the chambers and placing them back has likely caused disturbances. Apparently, it takes several minutes before CH_4_ production and emission are in equilibrium again. In a lab experiment Christiansen et al.^32^ found indeed a 35% reduction in the first flux after placing the chamber compared to a reference flux. The fact that not all first measurements after ventilating resulted in a discrepancy, can most probably be explained by the differences in time between ventilating and the measurement (between 1 and 15 min). Overall, comparing CH_4_ fluxes measured with the EC and the chamber method, we show similar results with respect to magnitude and diurnal patterns. We conclude that the chamber method yields representative CH_4_ fluxes for the studied ecosystem when fluxes within the first 10 min after ventilating are eliminated.

In summary, pressurized flow in *Phragmites* does seem to increase the total CH_4_ emission, even though ebullition is much reduced. It means that the effect of CH_4_ bypassing the oxic water layer due to plant transport is much larger than the effect of O_2_ transport by the plants on CH_4_ oxidation and production in the rhizosphere. This research was only covering a period within the growing stage of *Phragmites* with a high water table, giving a first indication of the contribution of the different flux pathways. To know the overall effect of the pressurized flow in *Phragmites* on CH_4_ emission, this experiment should be repeated to cover the whole growing season and variations in water table. Overall, this research emphasizes that plants with pressurized gas transport mechanism can be an important contributor to CH_4_ emission from wetlands.

## Data Availability

All data generated or analyzed during this study are included in this published article.
